# Bis[2-(1-imino­eth­yl)phenolato-κ^2^ *N*,*O*]nickel(II)

**DOI:** 10.1107/S1600536811051476

**Published:** 2011-12-03

**Authors:** Ning Wang

**Affiliations:** a Department of Chemical Engineering, Henan University of Urban Construction, Pingdingshan 467044, People’s Republic of China

## Abstract

There are one and a half independent mol­ecules in the asymmetric unit of the title compound, [Ni(C_8_H_8_NO)_2_], one of which is situated on an inversion center. In both mol­ecules, the Ni^II^ ion is coordinated by two O and two N atoms from two Schiff base ligands in an approximate square-planar geometry. Inter­molecular N—H⋯O hydrogen bonds link three mol­ecules into centrosymmetric trimer. The crystal packing exhibits weak inter­molecular C—H⋯O hydrogen bonds and voids of 37 Å^3^.

## Related literature

For general background to the use of Schiff bases in coord­in­ation chemistry, see: Haikarainen *et al.* (2001 ▶); Miyasaka *et al.* (2002 ▶). For nickel complexes with Schiff base ligands, see: Liu *et al.* (2006 ▶); Wang (2010 ▶). For the crystal structure of a similar copper(II) complex, see: Marongiu & Lingafelter (1971 ▶).
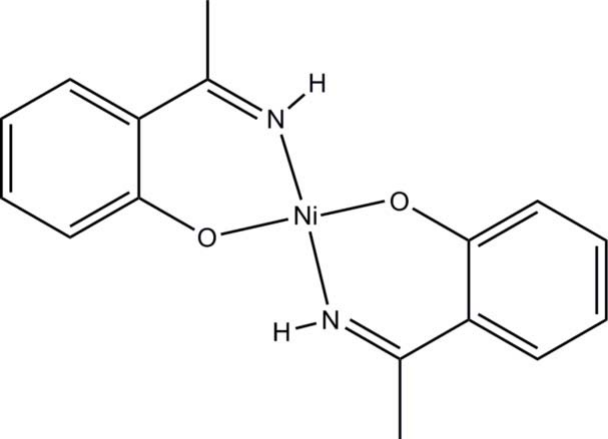


## Experimental

### 

#### Crystal data

[Ni(C_8_H_8_NO)_2_]
*M* *_r_* = 327.02
Triclinic, 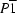

*a* = 9.1084 (10) Å
*b* = 11.3612 (16) Å
*c* = 11.8249 (18) Å
α = 101.006 (3)°
β = 93.049 (3)°
γ = 109.777 (3)°
*V* = 1121.1 (3) Å^3^
*Z* = 3
Mo *K*α radiation
μ = 1.30 mm^−1^
*T* = 298 K
0.20 × 0.20 × 0.18 mm

#### Data collection

Bruker APEXII CCD area-detector diffractometer
Absorption correction: multi-scan (*SADABS*; Sheldrick, 2004 ▶) *T*_min_ = 0.781, *T*_max_ = 0.799
6121 measured reflections
4190 independent reflections
2297 reflections with *I* > 2σ(*I*)
*R*_int_ = 0.044

#### Refinement

*R*[*F* ^2^ > 2σ(*F* ^2^)] = 0.062
*wR*(*F* ^2^) = 0.146
*S* = 0.99
4190 reflections
298 parameters
3 restraints
H atoms treated by a mixture of independent and constrained refinement
Δρ_max_ = 0.48 e Å^−3^
Δρ_min_ = −0.39 e Å^−3^

### 

Data collection: *APEX2* (Bruker, 2004 ▶); cell refinement: *SAINT* (Bruker, 2004 ▶); data reduction: *SAINT*; program(s) used to solve structure: *SHELXS97* (Sheldrick, 2008 ▶); program(s) used to refine structure: *SHELXL97* (Sheldrick, 2008 ▶); molecular graphics: *SHELXTL* (Sheldrick, 2008 ▶); software used to prepare material for publication: *SHELXTL*.

## Supplementary Material

Crystal structure: contains datablock(s) global, I. DOI: 10.1107/S1600536811051476/cv5207sup1.cif

Structure factors: contains datablock(s) I. DOI: 10.1107/S1600536811051476/cv5207Isup2.hkl

Additional supplementary materials:  crystallographic information; 3D view; checkCIF report

## Figures and Tables

**Table 1:** Hydrogen-bond geometry (Å, °)

*D*—H⋯*A*	*D*—H	H⋯*A*	*D*⋯*A*	*D*—H⋯*A*
N3—H3*A*⋯O2^i^	0.90 (1)	2.16 (2)	3.055 (6)	172 (6)
N1—H1⋯O3	0.90 (1)	2.25 (2)	3.138 (6)	168 (6)
C22—H22⋯O1^ii^	0.93	2.46	3.332 (6)	157 (6)

Symmetry codes: (i) 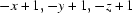; (ii) 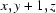.

**Fig. 1.**
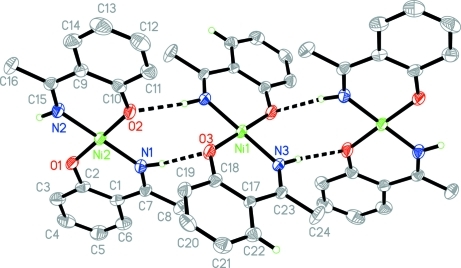
The hydrogen-bonded (dashed lines) trimer in (I), showing the atom-labelling scheme and displacement ellipsoids drawn at the 30% probability level. Unlabeled atoms are related to the labeled ones by the symmetry operation (1 - x, 1 - y, 1 - z).

## supplementary crystallographic information

### Comment

The Schiff bases are a kind of versatile ligands used in coordination chemistry (Haikarainen *et al.*, 2001; Miyasaka *et al.*, 2002). The complexes derived from Schiff bases have proved to be of significant interest in the areas of catalysis, magnetism, medicinal and material chemistry. In the present paper, the title compound (I) - a new Schiff base nickel(II) complex - is reported.

The molecule of (I) is mononuclear nickel(II) complex. The asymmetric unit of (I) contains  two crystallographically independent molecules, one of which is situated on inversion center. The Ni atom is coordinated by two O and two N atoms from two Schiff base ligands, forming a square planar geometry. The bond lengths related to the Ni atoms are comparable to those observed in other nickel(II) complexes with Schiff bases (Liu *et al.*, 2006; Wang, 2010), but shorter than the Cu–N and Cu–O bonds observed in a structurally similar copper(II) complex (Marongiu & Lingafelter, 1971).

Intermolecular N—H···O hydrogen bonds link three molecules in (I) into centrosymmetric trimer (Fig. 1). The crystal packing exhibits weak intermolecular C—H···O hydrogen bonds and voids of 37 Å^3^.

### Experimental

To a stirred ethanolic solution (30 ml) of 2-acetylphenol (0.136 g, 1 mmol) was added a few drops of 30% ammonia and an ethanolic solution (20 ml) of nickel(II) nitrate hexahydrate (0.291 g, 1 mmol). The final mixture was further stirred at room temperature for 1 h. The clear solution was set aside for a week, yielding red small block-shaped single crystals.

### Refinement

The amino H atoms were located in a difference Fourier map and were refined with distance restraints of N—H = 0.90 (1) Å. All other H atoms were positioned geometrically and were constrained as riding atoms, with C—H distances of 0.93–0.96 Å, and *U*_iso_(H) set to 1.2 or 1.5*U*_eq_(C) of the parent atom. Rotating group models were used for the methyl groups. The structure contains voids of 37 Å^3^.

### Crystal data

**Table:**

[Ni(C_8_H_8_NO)_2_]	*Z* = 3
*M**_r_* = 327.02	*F*(000) = 510
Triclinic, *P*1	*D*_x_ = 1.453 Mg m^−^^3^
*a* = 9.1084 (10) Å	Mo *K*α radiation, λ = 0.71073 Å
*b* = 11.3612 (16) Å	Cell parameters from 772 reflections
*c* = 11.8249 (18) Å	θ = 2.3–24.5°
α = 101.006 (3)°	µ = 1.30 mm^−^^1^
β = 93.049 (3)°	*T* = 298 K
γ = 109.777 (3)°	Block, red
*V* = 1121.1 (3) Å^3^	0.20 × 0.20 × 0.18 mm

### Data collection

**Table:**

Bruker APEXII CCD area-detector diffractometer	4190 independent reflections
Radiation source: fine-focus sealed tube	2297 reflections with *I* > 2σ(*I*)
graphite	*R*_int_ = 0.044
ω scans	θ_max_ = 25.7°, θ_min_ = 1.8°
Absorption correction: multi-scan (*SADABS*; Sheldrick, 2004)	*h* = −11→11
*T*_min_ = 0.781, *T*_max_ = 0.799	*k* = −13→13
6121 measured reflections	*l* = −13→14

### Refinement

**Table:**

Refinement on *F*^2^	Primary atom site location: structure-invariant direct methods
Least-squares matrix: full	Secondary atom site location: difference Fourier map
*R*[*F*^2^ > 2σ(*F*^2^)] = 0.062	Hydrogen site location: inferred from neighbouring sites
*wR*(*F*^2^) = 0.146	H atoms treated by a mixture of independent and constrained refinement
*S* = 0.99	*w* = 1/[σ^2^(*F*_o_^2^) + (0.0585*P*)^2^] where *P* = (*F*_o_^2^ + 2*F*_c_^2^)/3
4190 reflections	(Δ/σ)_max_ < 0.001
298 parameters	Δρ_max_ = 0.48 e Å^−^^3^
3 restraints	Δρ_min_ = −0.39 e Å^−^^3^

### Special details

**Table:**

Geometry. All e.s.d.'s (except the e.s.d. in the dihedral angle between two l.s. planes) are estimated using the full covariance matrix. The cell e.s.d.'s are taken into account individually in the estimation of e.s.d.'s in distances, angles and torsion angles; correlations between e.s.d.'s in cell parameters are only used when they are defined by crystal symmetry. An approximate (isotropic) treatment of cell e.s.d.'s is used for estimating e.s.d.'s involving l.s. planes.
Refinement. Refinement of *F*^2^ against ALL reflections. The weighted *R*-factor *wR* and goodness of fit *S* are based on *F*^2^, conventional *R*-factors *R* are based on *F*, with *F* set to zero for negative *F*^2^. The threshold expression of *F*^2^ > σ(*F*^2^) is used only for calculating *R*-factors(gt) *etc*. and is not relevant to the choice of reflections for refinement. *R*-factors based on *F*^2^ are statistically about twice as large as those based on *F*, and *R*- factors based on ALL data will be even larger.

### Fractional atomic coordinates and isotropic or equivalent isotropic displacement parameters (Å^2^)

**Table:**

	*x*	*y*	*z*	*U*_iso_*/*U*_eq_	
Ni1	0.5000	0.5000	0.5000	0.0392 (3)	
Ni2	0.35993 (9)	0.05065 (7)	0.23238 (6)	0.0385 (3)	
O1	0.1878 (5)	−0.0945 (3)	0.2135 (3)	0.0472 (11)	
O2	0.5346 (5)	0.1953 (3)	0.2545 (3)	0.0473 (11)	
O3	0.4558 (5)	0.4475 (3)	0.3430 (3)	0.0477 (11)	
N1	0.2586 (6)	0.1539 (4)	0.3058 (4)	0.0423 (12)	
N2	0.4618 (6)	−0.0507 (4)	0.1545 (4)	0.0423 (12)	
N3	0.4211 (6)	0.6312 (4)	0.5050 (4)	0.0416 (12)	
C1	0.0143 (7)	−0.0061 (6)	0.3199 (5)	0.0451 (15)	
C2	0.0540 (7)	−0.1072 (6)	0.2547 (5)	0.0445 (16)	
C3	−0.0595 (8)	−0.2330 (6)	0.2351 (6)	0.0571 (18)	
H3	−0.0379	−0.3009	0.1913	0.069*	
C4	−0.1993 (8)	−0.2566 (7)	0.2789 (6)	0.067 (2)	
H4	−0.2708	−0.3403	0.2651	0.080*	
C5	−0.2365 (9)	−0.1581 (8)	0.3436 (6)	0.070 (2)	
H5	−0.3316	−0.1752	0.3741	0.084*	
C6	−0.1317 (8)	−0.0350 (7)	0.3621 (6)	0.0594 (19)	
H6	−0.1581	0.0314	0.4039	0.071*	
C7	0.1213 (7)	0.1262 (6)	0.3398 (5)	0.0423 (15)	
C8	0.0716 (8)	0.2323 (6)	0.4004 (6)	0.065 (2)	
H8A	0.1536	0.3133	0.4047	0.098*	
H8B	0.0519	0.2216	0.4775	0.098*	
H8C	−0.0226	0.2298	0.3579	0.098*	
C9	0.7050 (7)	0.1099 (6)	0.1411 (5)	0.0419 (15)	
C10	0.6652 (7)	0.2101 (6)	0.2071 (5)	0.0433 (15)	
C11	0.7740 (8)	0.3369 (6)	0.2241 (6)	0.0563 (18)	
H11	0.7512	0.4047	0.2672	0.068*	
C12	0.9132 (9)	0.3605 (7)	0.1774 (6)	0.066 (2)	
H12	0.9839	0.4444	0.1908	0.080*	
C13	0.9520 (9)	0.2637 (9)	0.1111 (7)	0.075 (2)	
H13	1.0458	0.2816	0.0787	0.090*	
C14	0.8479 (8)	0.1411 (7)	0.0948 (6)	0.0613 (19)	
H14	0.8730	0.0752	0.0508	0.074*	
C15	0.5979 (7)	−0.0231 (5)	0.1195 (5)	0.0396 (15)	
C16	0.6453 (8)	−0.1295 (6)	0.0558 (5)	0.0563 (18)	
H16A	0.5654	−0.2106	0.0551	0.084*	
H16B	0.7430	−0.1254	0.0941	0.084*	
H16C	0.6577	−0.1204	−0.0226	0.084*	
C17	0.3474 (6)	0.6042 (5)	0.3019 (5)	0.0354 (14)	
C18	0.3980 (7)	0.4986 (5)	0.2679 (5)	0.0412 (15)	
C19	0.3859 (7)	0.4450 (6)	0.1502 (5)	0.0491 (16)	
H19	0.4205	0.3770	0.1271	0.059*	
C20	0.3235 (8)	0.4911 (6)	0.0672 (6)	0.0562 (18)	
H20	0.3155	0.4532	−0.0109	0.067*	
C21	0.2730 (8)	0.5925 (6)	0.0987 (6)	0.0567 (18)	
H21	0.2314	0.6234	0.0422	0.068*	
C22	0.2843 (7)	0.6477 (6)	0.2141 (6)	0.0474 (16)	
H22	0.2494	0.7159	0.2348	0.057*	
C23	0.3618 (6)	0.6684 (5)	0.4228 (5)	0.0368 (14)	
C24	0.3106 (9)	0.7818 (6)	0.4540 (5)	0.066 (2)	
H24A	0.3309	0.8143	0.5366	0.099*	
H24B	0.2000	0.7559	0.4291	0.099*	
H24C	0.3680	0.8477	0.4161	0.099*	
H1	0.306 (7)	0.2400 (13)	0.325 (5)	0.080*	
H2	0.399 (6)	−0.134 (2)	0.139 (5)	0.080*	
H3A	0.434 (8)	0.676 (5)	0.578 (2)	0.080*	

### Atomic displacement parameters (Å^2^)

**Table:**

	*U*^11^	*U*^22^	*U*^33^	*U*^12^	*U*^13^	*U*^23^
Ni1	0.0459 (7)	0.0321 (6)	0.0439 (7)	0.0225 (6)	0.0028 (6)	0.0035 (5)
Ni2	0.0378 (5)	0.0330 (5)	0.0473 (5)	0.0168 (4)	0.0084 (4)	0.0063 (4)
O1	0.038 (2)	0.038 (2)	0.067 (3)	0.018 (2)	0.012 (2)	0.006 (2)
O2	0.045 (3)	0.037 (2)	0.057 (3)	0.016 (2)	0.016 (2)	−0.001 (2)
O3	0.069 (3)	0.041 (2)	0.041 (3)	0.034 (2)	−0.001 (2)	0.003 (2)
N1	0.047 (3)	0.035 (3)	0.044 (3)	0.017 (3)	0.003 (3)	0.004 (3)
N2	0.043 (3)	0.036 (3)	0.049 (3)	0.015 (3)	0.015 (3)	0.006 (3)
N3	0.046 (3)	0.038 (3)	0.044 (3)	0.024 (3)	0.000 (3)	0.003 (3)
C1	0.036 (4)	0.054 (4)	0.050 (4)	0.022 (3)	0.004 (3)	0.014 (3)
C2	0.036 (4)	0.046 (4)	0.058 (4)	0.018 (3)	0.005 (3)	0.023 (3)
C3	0.044 (4)	0.053 (4)	0.076 (5)	0.016 (4)	0.003 (4)	0.020 (4)
C4	0.044 (5)	0.069 (5)	0.079 (6)	0.001 (4)	0.001 (4)	0.033 (4)
C5	0.048 (5)	0.088 (6)	0.078 (6)	0.024 (5)	0.020 (4)	0.025 (5)
C6	0.045 (4)	0.073 (5)	0.067 (5)	0.026 (4)	0.014 (4)	0.021 (4)
C7	0.049 (4)	0.053 (4)	0.032 (4)	0.031 (4)	0.004 (3)	0.005 (3)
C8	0.058 (5)	0.069 (5)	0.077 (5)	0.038 (4)	0.015 (4)	0.004 (4)
C9	0.039 (4)	0.047 (4)	0.042 (4)	0.019 (3)	0.004 (3)	0.008 (3)
C10	0.043 (4)	0.045 (4)	0.039 (4)	0.012 (3)	−0.002 (3)	0.010 (3)
C11	0.047 (4)	0.049 (4)	0.067 (5)	0.012 (4)	0.003 (4)	0.012 (4)
C12	0.054 (5)	0.065 (5)	0.068 (5)	0.001 (4)	0.005 (4)	0.023 (4)
C13	0.044 (5)	0.102 (7)	0.069 (6)	0.010 (5)	0.011 (4)	0.020 (5)
C14	0.042 (4)	0.080 (5)	0.062 (5)	0.024 (4)	0.013 (4)	0.011 (4)
C15	0.045 (4)	0.045 (4)	0.036 (4)	0.024 (3)	0.002 (3)	0.013 (3)
C16	0.066 (5)	0.056 (4)	0.059 (4)	0.038 (4)	0.020 (4)	0.010 (4)
C17	0.028 (3)	0.032 (3)	0.046 (4)	0.012 (3)	0.004 (3)	0.006 (3)
C18	0.036 (4)	0.038 (3)	0.051 (4)	0.015 (3)	0.011 (3)	0.011 (3)
C19	0.056 (4)	0.047 (4)	0.043 (4)	0.021 (3)	0.006 (3)	0.004 (3)
C20	0.058 (4)	0.059 (4)	0.040 (4)	0.010 (4)	−0.001 (4)	0.006 (4)
C21	0.058 (5)	0.059 (5)	0.056 (5)	0.019 (4)	0.001 (4)	0.026 (4)
C22	0.046 (4)	0.045 (4)	0.056 (5)	0.018 (3)	0.006 (3)	0.019 (3)
C23	0.032 (3)	0.030 (3)	0.050 (4)	0.013 (3)	0.010 (3)	0.007 (3)
C24	0.102 (6)	0.063 (4)	0.059 (5)	0.062 (5)	0.017 (4)	0.013 (4)

### Geometric parameters (Å, °)

**Table:**

Ni1—O3^i^	1.816 (4)	C8—H8C	0.9600
Ni1—O3	1.816 (4)	C9—C14	1.398 (8)
Ni1—N3	1.853 (5)	C9—C10	1.417 (8)
Ni1—N3^i^	1.853 (5)	C9—C15	1.459 (8)
Ni2—O1	1.817 (4)	C10—C11	1.414 (8)
Ni2—O2	1.822 (4)	C11—C12	1.374 (9)
Ni2—N1	1.847 (5)	C11—H11	0.9300
Ni2—N2	1.856 (5)	C12—C13	1.382 (10)
O1—C2	1.310 (7)	C12—H12	0.9300
O2—C10	1.315 (7)	C13—C14	1.364 (9)
O3—C18	1.326 (6)	C13—H13	0.9300
N1—C7	1.289 (7)	C14—H14	0.9300
N1—H1	0.902 (10)	C15—C16	1.501 (7)
N2—C15	1.283 (7)	C16—H16A	0.9600
N2—H2	0.899 (10)	C16—H16B	0.9600
N3—C23	1.294 (7)	C16—H16C	0.9600
N3—H3A	0.897 (10)	C17—C22	1.406 (7)
C1—C6	1.400 (8)	C17—C18	1.422 (7)
C1—C2	1.420 (8)	C17—C23	1.453 (7)
C1—C7	1.454 (8)	C18—C19	1.391 (8)
C2—C3	1.418 (8)	C19—C20	1.378 (8)
C3—C4	1.361 (9)	C19—H19	0.9300
C3—H3	0.9300	C20—C21	1.375 (8)
C4—C5	1.383 (9)	C20—H20	0.9300
C4—H4	0.9300	C21—C22	1.371 (8)
C5—C6	1.369 (9)	C21—H21	0.9300
C5—H5	0.9300	C22—H22	0.9300
C6—H6	0.9300	C23—C24	1.502 (7)
C7—C8	1.498 (8)	C24—H24A	0.9600
C8—H8A	0.9600	C24—H24B	0.9600
C8—H8B	0.9600	C24—H24C	0.9600
			
O3^i^—Ni1—O3	180.000 (1)	C10—C9—C15	120.8 (5)
O3^i^—Ni1—N3	86.81 (18)	O2—C10—C11	116.8 (6)
O3—Ni1—N3	93.19 (18)	O2—C10—C9	125.4 (5)
O3^i^—Ni1—N3^i^	93.19 (18)	C11—C10—C9	117.9 (6)
O3—Ni1—N3^i^	86.81 (18)	C12—C11—C10	120.3 (7)
N3—Ni1—N3^i^	180.000 (2)	C12—C11—H11	119.8
O1—Ni2—O2	178.57 (18)	C10—C11—H11	119.8
O1—Ni2—N1	93.24 (19)	C11—C12—C13	122.3 (7)
O2—Ni2—N1	87.0 (2)	C11—C12—H12	118.9
O1—Ni2—N2	87.43 (19)	C13—C12—H12	118.9
O2—Ni2—N2	92.39 (19)	C14—C13—C12	117.7 (7)
N1—Ni2—N2	178.2 (2)	C14—C13—H13	121.2
C2—O1—Ni2	127.8 (4)	C12—C13—H13	121.2
C10—O2—Ni2	127.8 (4)	C13—C14—C9	123.1 (7)
C18—O3—Ni1	129.2 (4)	C13—C14—H14	118.4
C7—N1—Ni2	131.3 (4)	C9—C14—H14	118.4
C7—N1—H1	108 (4)	N2—C15—C9	120.5 (5)
Ni2—N1—H1	121 (4)	N2—C15—C16	119.1 (5)
C15—N2—Ni2	132.2 (4)	C9—C15—C16	120.4 (5)
C15—N2—H2	118 (4)	C15—C16—H16A	109.5
Ni2—N2—H2	110 (4)	C15—C16—H16B	109.5
C23—N3—Ni1	131.0 (4)	H16A—C16—H16B	109.5
C23—N3—H3A	119 (4)	C15—C16—H16C	109.5
Ni1—N3—H3A	110 (4)	H16A—C16—H16C	109.5
C6—C1—C2	119.1 (6)	H16B—C16—H16C	109.5
C6—C1—C7	120.2 (6)	C22—C17—C18	118.0 (5)
C2—C1—C7	120.6 (5)	C22—C17—C23	119.9 (5)
O1—C2—C3	117.3 (6)	C18—C17—C23	122.1 (5)
O1—C2—C1	125.5 (5)	O3—C18—C19	117.8 (5)
C3—C2—C1	117.2 (6)	O3—C18—C17	123.3 (5)
C4—C3—C2	121.7 (7)	C19—C18—C17	118.9 (5)
C4—C3—H3	119.2	C20—C19—C18	121.1 (6)
C2—C3—H3	119.2	C20—C19—H19	119.5
C3—C4—C5	121.0 (7)	C18—C19—H19	119.5
C3—C4—H4	119.5	C21—C20—C19	120.8 (6)
C5—C4—H4	119.5	C21—C20—H20	119.6
C6—C5—C4	119.2 (7)	C19—C20—H20	119.6
C6—C5—H5	120.4	C22—C21—C20	119.4 (6)
C4—C5—H5	120.4	C22—C21—H21	120.3
C5—C6—C1	121.9 (7)	C20—C21—H21	120.3
C5—C6—H6	119.0	C21—C22—C17	121.9 (6)
C1—C6—H6	119.0	C21—C22—H22	119.1
N1—C7—C1	121.0 (5)	C17—C22—H22	119.1
N1—C7—C8	119.3 (6)	N3—C23—C17	121.1 (5)
C1—C7—C8	119.7 (6)	N3—C23—C24	118.8 (5)
C7—C8—H8A	109.5	C17—C23—C24	120.1 (5)
C7—C8—H8B	109.5	C23—C24—H24A	109.5
H8A—C8—H8B	109.5	C23—C24—H24B	109.5
C7—C8—H8C	109.5	H24A—C24—H24B	109.5
H8A—C8—H8C	109.5	C23—C24—H24C	109.5
H8B—C8—H8C	109.5	H24A—C24—H24C	109.5
C14—C9—C10	118.7 (6)	H24B—C24—H24C	109.5
C14—C9—C15	120.5 (6)		

Symmetry codes: (i) −*x*+1, −*y*+1, −*z*+1.

### Hydrogen-bond geometry (Å, °)

**Table:**

*D*—H···*A*	*D*—H	H···*A*	*D*···*A*	*D*—H···*A*
N3—H3A···O2^i^	0.90 (1)	2.16 (2)	3.055 (6)	172 (6)
N1—H1···O3	0.90 (1)	2.25 (2)	3.138 (6)	168 (6)
C22—H22···O1^ii^	0.93	2.46	3.332 (6)	157 (6)

Symmetry codes: (i) −*x*+1, −*y*+1, −*z*+1; (ii) *x*, *y*+1, *z*.
